# Impact of Financial Development on Income Gap Based on Improved Gaussian Kernel Function and BGP Anomaly Detection

**DOI:** 10.1155/2022/1064657

**Published:** 2022-09-19

**Authors:** Yan Yao, Yan Huang

**Affiliations:** School of Finance, Guangdong University of Finance & Economics, Guangzhou 510320, Guangdong, China

## Abstract

Nuclear methods, such as the study of the main components of nuclear and the support of vector machines, have gradually evolved into a type of pillar methods for pattern recognition and economic statistics. Therefore, how to choose the inner product function of high-dimensional space is particularly important for the use of such methods. And, because the inner product function of high-dimensional space often determines the structure of high-dimensional space, the superiority of the kernel method is directly affected by using high-dimensional space. Due to its superior performance, the inner product function of Gaussian high-dimensional space has been put into practical use to a large extent. For the processing of unbalanced distribution of BGP data, because the results of BGP usually have multiple results, and the distribution is not uniform, so in the process of training SVM, the process of determining the composition of the hyperplane is generated due to various results The effect of the results will also be different so that the final planning equation has errors and weakens the classification ability. Finally, the BGP test results are displayed. The results show that the operation of the SVM algorithm can complete the supervision and management of BGP abnormal data. If there is any data fluctuation, an alarm will be issued. Under the condition of ensuring the correct rate, the input characteristics of BGP can be reduced in dimensionality, and specific selection methods can be used to complete. A high-level algorithm is used here, so that it has a better SVM classification effect, and abnormal traffic can be more conveniently and quickly checked out of BGP. It can be seen from the results of statistical analysis of urban and rural data that the current gap between urban and rural economic development level is too large, and to some extent, there is a trend of increasing fault, which will undoubtedly affect the stability of urban and rural areas, so the urban-rural income gap should be narrowed. We have also studied the principle of the general preferential economy in rural areas and put forward relevant effective strategy.

## 1. Introduction

With the continuous development of the economy and the gradual implementation of industrialization, the urban economy supported by the secondary and tertiary industries has developed rapidly [1]. In contrast, rural areas continue the traditional production mode of primary industry, so the economic development level of urban and rural areas gradually appears a gap, and the income gap of residents is constantly widening [2]. It can be seen from the research that the urban-rural income gap has been continuously increasing since the 21st century. In 2000, the per capita disposable economic gap between urban and rural residents across the country could reach 3897.9 yuan, which has increased to 24634.0 yuan, and the expansion rate reached 516.17%. Therefore, how to choose the inner product function of high-dimensional space is particularly important for the use of such methods, and because the inner product function of high-dimensional space often determines the structure of high-dimensional space, the superiority of the kernel method is directly affected by the use of high-dimensional space [3]. The role of. Therefore, the inner product function of Gaussian high-dimensional space has been put into practical use to a large extent due to its superior performance. By constructing a model of the manufacturing function of the rural financial sector and the urban financial sector, it shows that the economic level gap between urban and rural is too large, and the distance between urban and rural residents' per capita income levels will also increase [4]. Therefore, it is necessary to make every effort to promote the development of the economic foundation of the countryside, so as to gradually reduce the economic distance between urban and rural areas, thereby gradually reducing the income gap between urban and rural areas [5]. We have studied the working principle of the ordinary preferential economy in rural areas and proposed relevant effective strategies.

## 2. Related Work

According to the literature, the current urban-rural income gap in China has been gradually existing since the founding of New China [6]. Literature has studied the industrial development model of New China and proposed that after the founding of China, the economic development based on the heavy industry was vigorously carried out in urban areas, while the rural areas were still in the traditional agricultural development model, which formed the difference between the two [7]. Literature suggests that the market economy business model brought about by urban economic development has further increased the gap between urban and rural areas [8]. Finance, as an important means of allocating scarce resources, is improving market efficiency and promoting economic growth. At the same time, because of its “dislike the poor and love the rich” characteristics, the distribution of resources appears unbalanced. Literature pointed out that financial development will bring changes to the urban-rural income gap and the development of finance will narrow the urban-rural income gap, so this article will discuss these issues [9]. We need to improve the financial model, and the social security system also needs to be reformed. In general, first, the monopoly level formed in the market is China's many state-owned companies. The participation and competition of these state-owned companies hinder healthy competition among various industries and is not conducive to the reasonable division of resources in the market. Literature specifically pointed out that for many poor areas, because there are not many companies, it is easy to be at a disadvantage in the competition, and there is no way to get valuable resources, so that income cannot grow and cannot be reduced [10]. The income level gap between regions. Therefore, China must seriously reform state-owned enterprises, improve their industrial chains, and improve their development difficulties. Second, China's social security system needs to be improved by relevant agencies, to increase the degree of economic support for social security in developing regions, to improve the systems in these regions, and to develop the financial industry. Literature pointed out that in order to fundamentally solve the related problems of income imbalance in some areas of China, the distribution system model still needs us to deepen the reform, which is beneficial to guarantee the stable and rapid economic development of various industries in China [11]. The literature proposes two countermeasures. One is to adopt a combination of party and government orders and legal constraints to severely punish economic activities that do not follow the development model, which makes the factors in China's financial development increasingly ideal. Let the concept of economic development of this model take root in the hearts of the people, rely on their own ability and level to reasonably obtain wealth [12]. The second is to appropriately increase the value of property in accordance with taxation and fiscal policies, and at the same time, it is necessary to restrict people with high income levels to a certain extent, so as to reasonably improve the problem of unequal distribution in the capital acquisition, literature mentioned in the protection of the effective monitoring and feedback of a small number of people on the overall national income, which is beneficial to the stability and prosperity of the entire country, ensures the steady development of the society, and lays a solid foundation for the construction of a harmonious social atmosphere in China [13].

## 3. Research on Improving the Inner Product Function of Gaussian High-Dimensional Space

For the influencing factors of classification, the situation that the linear classification method cannot be used to solve the problem often occurs, so it is necessary to adopt nonlinear classification techniques to solve the problem. Nuclear methods, such as the study of the main components of nuclear and the support of vector machines, have gradually evolved into a type of pillar methods for pattern recognition and economic statistics. Therefore, how to choose the inner product function of high-dimensional space is particularly important for the use of this kind of method. The nonlinear research equipment used in the old model is to directly study the initial data, which has serious limitations in the classification model. Compared with the old general classification equipment, the nuclear method has obvious advantages: (1) the nonlinear correspondence of is specific to specific practices, which facilitates the integration of problems related to the problem of early verification and can make up for the insensitive response of general nonlinear research equipment; (2) nonlinear research equipment has a control over linear research equipment. The research on the fitting phenomenon is more inaccurate, so the generalization ability cannot be guaranteed; (3) the implicit conversion of the kernel method makes the high-dimensional operation space independent of the computational complexity, thus realizing extremely sensitive calculations.

There are still many problems in the existing kernel methods, so this paper needs to put forward targeted solutions. This paper chooses the method of introducing the inner product function of high-dimensional space. The following is the explanation of the inner product function of the high-dimensional space. There may be a transformation of Hilbert space H.

(1) $$\begin{array}{c} R^{n}⟶H\;x⟶\phi \left(x\right). \end{array}$$

The form of the inner product function of the high-dimensional space determines the structure of the sample mapping to the high-dimensional feature space, so the inner product function of the high-dimensional space is of great significance to the kernel method. Because of the variety of inner product functions in high-dimensional spaces, it is an important issue to determine whether a function can be used as an inner product function in high-dimensional spaces. Generally speaking, as long as Mercer's condition is satisfied, we can call it the inner product function of high-dimensional space.

In terms of categories, there is a vector machine theory in the category of equipment created for nuclear method research. In addition, nuclear methods can penetrate regression, manifold, feature selection, clustering, nuclear Fisher discriminant analysis, and nuclear principal components. Analysis and many other aspects. Because it has a rigorous practical foundation and very efficient practical application performance, the application of nuclear methods has a very broad future.

### 3.1. Learning of Inner Product Function in High-Dimensional Space

Among the inner product functions of many high-dimensional spaces, the inner product function of Gaussian high-dimensional space has been widely used due to its good performance. In most situations, before using the inner product function of a high-dimensional space, we do not know whether the sample can be linearly divided under the action of the inner product function of the high-dimensional space, so the difference performance of the Gaussian kernel function is only It will be so strong, it will make it easier to extract some features of the sample.

### 3.2. Nuclear Target Measurement Criteria

The concept of nuclear objective measurement was first proposed by Cristianini and his team. For the inner product function of a high-dimensional space, given the inner product function of two high-dimensional spaces, calibrate and estimate the corresponding kernel matrix based on past experience. Connectivity, mathematically it is defined as follows:

(2) $$\begin{array}{c} A\left(K_{1},K_{2}\right)=\frac{{\langle K_{1},K_{2}\rangle }_{F}}{\sqrt{{\langle K_{1},K_{1}\rangle }_{F}{\langle K_{2},K_{2}\rangle }_{F}}}\text{.} \end{array}$$

Among them, k and A represent the inner product function of the high-dimensional space and the corresponding kernel matrix, and <&, &> is the Frobenius product between two matrices, which is defined as follows:

(3) $$\begin{array}{c} {\langle K_{1},K_{2}\rangle }_{F}=\overset{l}{\underset{i=1}{\sum }}\overset{l}{\underset{j=1}{\sum }}k_{1}\left(x_{i},x_{j}\right)k_{2}\left(x_{i},x_{j}\right)\text{.} \end{array}$$

If the kernel matrix is considered to be a two-dimensional vector, then this principle can be regarded as a cosine value for an angle. Therefore, the kernel matrix and the purpose matrix can be written in the following form:

(4) $$\begin{array}{c} \begin{array}{c} A\left(K_{1},K_{2}\right)=\frac{{\langle K,yy^{T}\rangle }_{F}}{\sqrt{{\langle K,K\rangle }_{F}{\langle yy^{T},yy^{T}\rangle }_{F}}}\\ =\frac{{\langle K,yy^{T}\rangle }_{F}}{l\sqrt{{\langle K,K\rangle }_{F}}}=\frac{y^{T}Ky}{l{\left\|K\right\|}_{F}} \end{array}\text{.} \end{array}$$

It has been confirmed that this method has many practical practical foundations: (1) computationally efficient: it only uses training samples and has a very efficient algorithm. Any computationally intensive nuclear training device is inferior to its time complexity. (2) Collectiveness: collectiveness means that it can be limited within a certain distance from the mean according to the amount of income obtained. Its expected value is highly concentrated near the nuclear calibration, so its experience value is stable for the different data obtained. This shows that verification calibration is based on empirical evaluation, and the possibility of real modification values that are very close is very high. (3) Enlargement capability: enlargement capability means the error rate or accuracy rate of classification when testing machines for various classification purposes. If the average value of the nuclear calibration is high, the value can be divided, and its magnification error rate can be reduced to an extremely low level. In addition, at the graphic level, finding a high-dimensional inner product function from a restricted high-dimensional space inner product function is a maximization goal of kernel calibration. The standardized ideal target matrix is

(5) $$\begin{array}{c} A\left(K,K*\right)=\left\|\frac{K}{{\left\|K\right\|}_{F}}-\frac{yy^{T}}{{\left\|yy^{T}\right\|}_{F}}\right\|=\sqrt{2-2A\left(K,K*\right)}\text{.} \end{array}$$

Online research algorithms have good characteristics such as simplicity, efficiency, and statistical assumptions that require only a few steps. These allow them to be practiced in a wide range of categories. Now consider the following problem of minimizing the objective function:

(6) $$\begin{array}{c} Q\left(\omega \right)=\overset{n}{\underset{i=1}{\sum }}Q_{i}\left(\omega \right)\text{.} \end{array}$$

The minimum value of the equation is generally achieved by performing multiple method simulations to obtain an optimal solution. The solution method is shown in the following equation:

(7) $$\begin{array}{c} \omega :=\omega -\eta \nabla Q\left(\omega \right)=\omega -\eta \overset{n}{\underset{i=1}{\sum }}\nabla Q_{i}\left(\omega \right)\text{.} \end{array}$$

Among them, *η* is the learning speed. In most cases, the function that requires the summation has a simple formula, which will make the calculation of the sum and stage of the summation function not very time-consuming, such as in multiparameter algorithms and statistics. To solve this problem, in each iteration, the online learning method only needs one sample, which is very useful for large-scale mechanical task learning. In the online research of the network, the gradient of a single sample is expressed as the similar value of the gradient of the objective function. The formula for the replacement is shown in the following equation:

(8) $$\begin{array}{c} \omega :=\omega -\eta \nabla Q\left(\omega \right)=\omega -\eta \overset{n}{\underset{i=1}{\sum }}\nabla Q_{i}\left(\omega \right)\text{.} \end{array}$$

In this online procedure, the samples to be tested are subjected to intensive calculations, only one sample is used for each change, and only the current single sample to be tested error rate gradient is considered when updating the parameters. In the batch program, all samples to be tested must be referenced for each replacement. When the sample base is large, this often becomes a very time-consuming step, but the online learning program can perform real-time based on a sample being tested update.

## 4. BGP Abnormal Detection

### 4.1. Research Status of BGP

In the recent period, the anomaly detection system mainly adopts methods such as statistical behavior, high-level participation, identification matching, and memory transfer, through event audit record analysis and pattern recognition model comparison, specific reports, and final results construction. Due to the continuous changes in attack methods, these anomaly detection systems are difficult to control due to their hand-coded characteristics. Therefore, stronger and more stable methods are needed to test for improper behavior. BP neural network is a neural sequence based on n error diffusion algorithms and can be reflected in many aspects and has the ability to learn and adapt to instability. And, can well meet the needs of scoring and competition recognition, due to its uniqueness and diversity, each type of structured data can be processed and has the function of automatic processing; in an unfamiliar network environment, you can establish a corresponding by inputting the test program, aiming at the detection of anomalies, and using the passed algorithm to maximize the unknowns of the member equation. The component function parameters related to the ordered parameters shall be combined and coded as a single general unknown number. Integrate different data into individual generic development to find the best unknown result. The first to carry out the analysis and research work on the BGP update information of the Internet elite network was Labovitz et al., who explained the results. The author found that a large amount of pathological routing information was generated by the Internet domain routing system and carried out a review. A series of studies have also discovered the main factors leading to pathological routing information.

Lad et al. used the U algorithm to make the amount of information carried on each connection line the rank of the line, but they mainly adopted the algorithmic skills of a specific scheme but only used partial replacement results of the line rank, and they could not classify themselves. When the Naive Bayesian method is being used to construct the anomaly test panel, for small-scale technical training, the appropriate data are accurately extracted. It has a definite use value. An effective entry test mode based on learning and strengthening rules is used in all preestimated targets. Adjust the update process of strengthening strategy weights in order to weaken the result of the recession. Then modify the old rule learning algorithm, and finally, after a certain improvement of the Boosting algorithm, it is used to strengthen the weakening ability of the rule research equipment on the network audit data.

### 4.2. BGP Protocol and BGP Abnormal Traffic Detection

All Internet is very convenient. In order to improve the operation speed of the management interdomain routing system, it is attributed to several independent management levels. Each part of the Internet is called a self-management department. The initial meaning of the self-management department is that the intradomain routing protocols are jointly undertaken and run together, a group of drives with the same rotation parameters under the same technology, and the external rotation of the interactive rotation protocol is a unified whole. However, the key point is that the rotation of the self-restraint program is carried out under the rotation arrangement plan that obeys the instructions, which reflects the continuity and compliance of the rotation.

**Figure 1:**
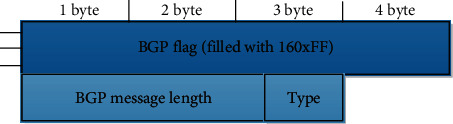
Self-management department diagram.

As shown in Figure 1, the layering of the Internet in the routing architecture determines that the mobile port of the Internet can also have a two-layer topology. One of them is the high-level self-determination management level department. They are the real topology, that is, the domain link rotation between the nodes in its own management department and the self-management department are all constructed by domain links. Another topology. The structure is a low-level rotation level, in which there is the node topology in the router, and the physical routing topology between the control panels is the physical connection.

**Figure 2:**
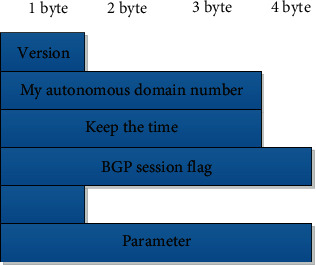
BGP header format.

The IETF working group respectively announced version 1 of the BGP protocol in 1989, version 2 in 1990, and version 3 in 1993. The current version is version 4 on the market. The recommendation number is RFC1771. The BGP protocol mentioned in this article. In fact, it is BGP-4. BGP header format as shown in Figure 2.

The open message format used to mark the version of BGP is usually the first byte, and it is now the fourth version. The last two letters are often used to record the sender's autonomous domain number, the next two letters are used to negotiate the set of the maximum waiting time between two users or updated peers during a BGP session, and the fourth letter is used to record the person who sent the message, the designated BGP field of the advertiser logo is filled with the p address of BGP, and the last letter is used to fill in the parameter open message distance.

**Figure 3:**
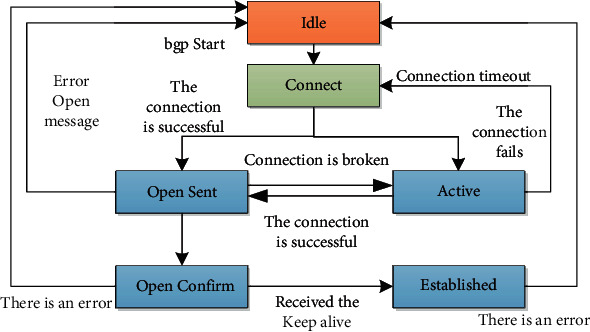
NOTIFICATION message format.

The message form of KEEPALIVE has only the header message. The error code is a letter to indicate the type of error. It is used to mark a more detailed error type, which is represented by the error subcode of a letter. The characteristics of a code sequence It is composed of a long sequence that can be changed, and the complete sequence is determined according to the subcode and error code of the error. The message sequence of NOTIFICATION is a 21+ length data. NOTIFICATION message format as shown in Figure 3.

### 4.3. BGP Protocol Working Process

TCP's contract runs under BGP, using TCP client 179 as its transmission information end. Create a reliable information delivery system from TCP, and exchange information in the neighbors of BGP, before BGP exchanges information at the same point, BGP first establishes a reliable transmission mechanism. Adjacent GBP peers are configured as special entry addresses, not neighbors that are searched under changing states. After BGP establishes a neighbor relationship subsequently, BGP uses regular keep alive messages to determine the availability of BGP neighbors.

Through abnormal testing, the data collected from several key points in the computer network or computer system are processed and screened to further detect whether there are violations of security measures and traces of being attacked on the network or system. This article mainly explains whether BGP abnormal traffic is caused by the spread of viruses. Although viruses that have not directly affected BGP routers are spreading, the CPU, internal storage overload, and defective routers in contract implementation are their faults. The main reason is that the routers and BGP of the entire Internet have been greatly damaged. These are enough to show that the hidden dangers and problems at the traffic level and personnel allocation level have a huge impact on the control plane. This article is based on the characteristics of BGP abnormal data. According to BGP, the traffic pattern shown by irregular sequences such as viruses in unusual forms is different from the usual situation. Using SVM, a part of the sequence is used as a sample. The test is performed to generate the SVM classifier, which requires the synthesis of the training program sequence to test the suspicious data of the estimated BGP.

## 5. Analysis of the Gap between China's Economic Development and the Wealth Value of Residents' Income

### 5.1. The Duality of the Wealth Value of Chinese Residents' Income

**Figure 4:**
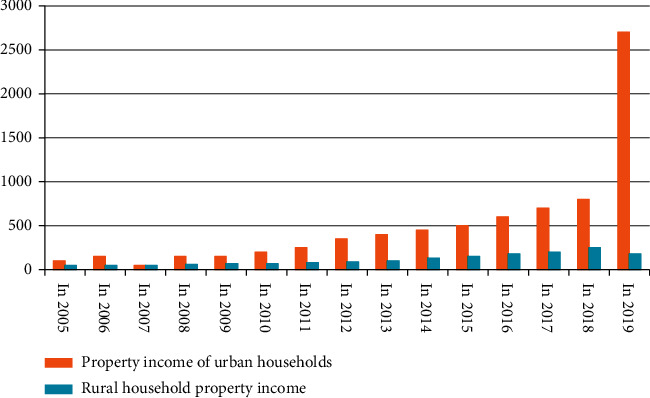
Total wealth value per capita annual income of urban and rural households in China (yuan).

In recent years, property income has been increasing year by year. The absolute value of property income of urban and rural residents in China has increased year by year. In 1990, the value of the wealth of urban residents was even lower than that of rural residents. By 1995, the per capita income of rural residents The wealth value of income reached 0.66 times that of urban residents. For the next 20 years, this data remained high, reaching a peak value of 4.38 in 1998. Obviously, the wealth value distance of income between towns and villages is gradually increasing, and at the same time the “Matthew effect” is revealed. Total wealth value per capita annual income of urban and rural households in China as shown in Figure 4. Comparison of Wealth Value Scale of Per Capita Income of Urban and Rural Households as shown in Table 1.

**Figure 5:**
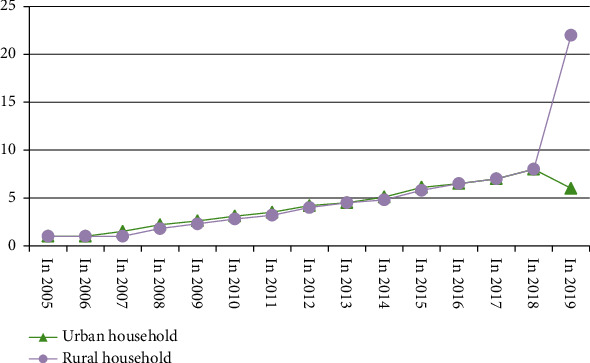
Comparison of the growth trend of wealth value per capita income of urban and rural residents.

The development of urban and rural economy in recent years can be seen from Figure 5, while the growth of annual average income of urban and rural households can be compared and analyzed from Table 2.

The continuous development and improvement of the financial market enables low-income earners to increase their income by taking advantage of newly emerging investment opportunities, thereby helping to alleviate the further widening of the income gap. Law et al. studied the influence of the system on the relationship between financial development and income disparity, and the research showed that only by breaking through the system, economic development can help reduce the distance between incomes. According to the cross-sectional data of the country in 2001, Liu Minlou used the scale of economic enterprises to reflect economic development and researched their impact on the gap in urban economic levels to expand and then decrease, showing an inverted “U” shape.

### 5.2. Model Construction and Interpretation

The production output of a country or region in a certain period is mainly determined by production technology, capital, and labor, as shown in the following equation:

(9) $$\begin{array}{c} Y_{t}=K_{t}^{0}{\left(A_{t}L_{t}\right)}^{1-\theta }\#\text{.} \end{array}$$

At present, China's urban and rural development presents an obvious dual pattern, and with the continuous economic development, the gap is increasing. Therefore, this paper designs two economic development models, rural and urban, as shown in formulas (10) and (11).

(10) $$\begin{array}{c} Y_{rt}=K_{rt}^{\alpha }{\left(A_{rt}L_{rt}\right)}^{f-\alpha }\text{,} \end{array}$$

(11) $$\begin{array}{c} Y_{ut}=K_{ut}^{\beta }{\left(A_{ut}L_{ut}\right)}^{f-\beta }\#\text{.} \end{array}$$

*K *_*rt*_ and *K*_*ut*_, respectively, represent the capital input of the rural economic sector and the urban economic sector in the t period, and Art and Aut represent the technological development level of the rural economic sector and the urban economic sector in the t period, respectively, and *L*_*rt*_ and Lut, respectively, represent the rural economy in the t period. The labor input of the sectors and urban economic sectors, *α* and *β* are parameters, 0 < *α*, *β* < 1, representing the marginal rate of return of rural capital and urban capital, respectively, and its magnitude depends on the economic impact of urban and rural economic sectors. Importance. Moreover, the increase in capital investment in urban economic sectors and rural economic sectors is shown in (12) and (13), respectively.

(12) $$\begin{array}{c} {\dot{K}}_{rt}=b_{r}L_{rt}K_{rt}\left(K_{ut}-K_{rt}\right)\text{,} \end{array}$$

(13) $$\begin{array}{c} {\dot{K}}_{ut}=b_{u}L_{ut}K_{ut}\#\text{.} \end{array}$$

At present, China has the policy means of economic regulation. If the gap between urban and rural economic development is too large, the rural economic policy will be optimized and the rural economic development will be more convenient. We will use policy measures to balance the development gap between urban and rural areas and prevent the imbalance of economic development between urban and rural areas. In view of the fact that the financial development of the rural financial sector and the urban financial sector are quite different, the imbalance of urban and rural financial development can be expressed as follows.

(14) $$\begin{array}{c} F=\frac{K_{ut}}{K_{rt}}\#\text{.} \end{array}$$

*Y *_*rt*_ and *Y*_*ut*_ obtain partial derivatives of *L*_*rt*_ and *L*_*ut*_, respectively, to obtain the income compensation *W*_*rt*_ and *W*_*ut*_ of rural and urban labor, as shown in (15) and (16), respectively.

(15) $$\begin{array}{c} W_{rt}=\frac{\partial Y_{rt}}{\partial L_{rt}}=\left(1-\alpha \right)K_{rt}^{\alpha }A_{rt}^{1-\alpha }L_{rt}^{-\alpha }\text{,} \end{array}$$

(16) $$\begin{array}{c} W_{ut}=\frac{\partial Y_{ut}}{\partial L_{ut}}=\left(1-\beta \right)K_{ut}^{\beta }A_{ut}^{1-\beta }L_{ut}^{-\beta }\#\text{.} \end{array}$$

*W *_*rt*_ and *W*_*ut*_ seek partial derivatives of *K*_*rt*_ and *K*_*ut*_, respectively, to clarify the impact of financial development on the income of rural residents and urban residents (17) and (18) are obtained.

(17) $$\begin{array}{c} \frac{\partial W_{rt}}{\partial K_{rt}}=\frac{\left(1-\alpha \right)\alpha A_{rt}^{1-\alpha }}{K_{rt}^{1-\alpha }L_{rt}^{\alpha }}>0 \end{array}$$

(18) $$\begin{array}{c} \frac{\partial W_{ut}}{\partial K_{ut}}=\frac{\left(1-\beta \right)\beta A_{ut}^{1-\beta }}{K_{ut}^{1-\beta }L_{ut}^{\beta }}>0\# \end{array}$$

Because 0<*α*<1, the derivation results are all greater than 0, indicating that for rural residents and urban residents, increasing the level of financial development can increase their income. In order to obtain the relationship between the income gap between urban and rural residents and financial development, the income gap between urban and rural residents is expressed as the ratio of urban and rural income. After calculation and sorting, the result is shown in the following equation:

(19) $$\begin{array}{c} Gap=\frac{W_{ut}}{W_{rt}}=\frac{\left(1-\beta \right)K_{ut}^{\beta }A_{ut}^{1-\beta }L_{ut}^{-\beta }}{\left(1-\beta \right)K_{rt}^{\alpha }A_{rt}^{1-\alpha }L_{rt}^{-\alpha }}\\ =\left(\frac{1-\beta }{1-\alpha }\right){\left(\frac{K_{ut}}{K_{rt}}\right)}^{\alpha }{\left(\frac{L_{ut}}{L_{rt}}\right)}^{-\alpha }{\left(\frac{K_{ut}}{L_{ut}}\right)}^{\beta -\alpha }\left(\frac{A_{ut}^{1-\beta }}{A_{rt}^{1-\alpha }}\right)\\ =\left(\frac{1-\beta }{1-\alpha }\right){\left(F\right)}^{\alpha }{\left(\frac{L_{ut}}{L_{rt}}\right)}^{-\alpha }{\left(\frac{K_{ut}}{L_{ut}}\right)}^{\beta -\alpha }\left(\frac{A_{ut}^{1-\beta }}{A_{rt}^{1-\alpha }}\right)\#. \end{array}$$

0 < *α*, *β* < 1, formula (11) shows that the greater the difference in financial development between urban and rural areas, the greater the income gap between urban and rural residents. This means that finance must be developed to benefit the rural economic sector.

### 5.3. Recommendations

For the government, it needs to make corresponding system revisions and policy adjustments, so as to provide the greatest possibility for diversified inclusive financial service innovation. The development of inclusive finance is inseparable from the government's creation of a good institutional environment. There are specific institutional arrangements under the institutional environment. The continuous expansion of the boundaries of old finance and the development of inclusive finance will inevitably cause relevant as the institutional arrangements change, there will be an incompatibility between the new institutional arrangements and the original institutional arrangements, and inappropriate institutional arrangements need to be reformed. Of course, the degree of regulatory slack is a test of the wisdom of the regulatory authorities. The government must weigh risks and innovations. It must not allow freedom or give up food due to choking. It should encourage innovation in inclusive finance within acceptable risks.

In addition, the government should also provide special care and support for inclusive financial products and innovative industries. For financial organizations, old financial intermediaries need to reform their organizational structure, and emerging financial intermediaries should overcome the deficiencies of old financial intermediaries in serving disadvantaged customers and innovate in service models. Financial organizations must also make full use of technological means to launch new products suitable for customers. Compared with the old financial products, the main body of inclusive financial supply must carry out various forms of product innovation, and exert the support for economic business in the era of big data.

## 6. Conclusion

However, the performance of this type of method greatly depends on the choice of the inner product function of the high-dimensional space. The inner product function of the high-dimensional space implicitly determines the value, and any two samples are concentrated in the inner product of the high-dimensional feature space. It provides a solid foundation for core-based learning methods in nonlinear analysis. The development of inclusive finance is inseparable from the government's creation of a good institutional environment. There are specific institutional arrangements under the institutional environment. The continuous expansion of the boundaries of old finance and the development of inclusive finance will inevitably cause relevant as the institutional arrangements change, there will be an incompatibility between the new institutional arrangements and the original institutional arrangements, and inappropriate institutional arrangements need to be reformed. The most challenging aspect of the kernel method is to determine a suitable inner product function of a high-dimensional space for a given data set. Finance is the core of the modern economy. The development of the modern economy cannot be separated from the support of finance. Financial development is promoting; at the same time of economic growth, it also provides an opportunity for individuals to use capital elements to improve their economic conditions. This article builds a production function model for rural economic sectors and urban economic sectors and finds that the greater the difference in financial development between urban and rural areas, the greater the income gap between urban and rural residents. Therefore, we should improve the poverty reduction effect of the economic situation and the trickle-down effect of getting rich first and getting rich later.

## Figures and Tables

**Table 1:** Comparison of wealth value scale of per capita income of urban and rural households in china.

Years	Urban residents	Rural residents	Urban rural gap coefficient
1990	15.60	28.96	0.54
1995	90.43	40.98	2.21
2000	128.38	45.04	2.85
2001	134.62	46.97	2.87
2002	102.12	50.68	2.01
2003	134.98	65.75	2.05
2004	161.15	76.61	2.10
2005	192.91	88.45	2.18
2006	244.01	100.50	2.43
2007	348.53	128.22	2.72
2008	387 02	148.08	2.61
2009	431.84	167.20	2.58
2010	520.33	202.25	2.57
2011	649.00	228.57	2.84
2012	706.96	249.05	2.84
2013	809.90	293.00	2.76
2014	2812.10	248.00	12.66

**Table 2:** Comparison of the annual growth rate of wealth value per capita income of urban and rural households (%).

Years	Urban residents			Urban residents		
	Amount (yuan)	Growth rate		Amount (yuan)	Growth rate	
		Growth rate (%)	Amplitude (%)		Growth rate (%)	Amplitude (%)
2000	128.38	1.00	−0.21	45.04	1.00	42.76
2001	134.62	1.05	4.86	46.97	1.04	4.29
2002	102.12	0.80	−24.14	50.68	1.13	7.90
2003	134.98	1.05	32.18	65.75	1.46	29.74
2004	161.15	1.26	19.39	76.61	1.70	16.52
2005	192.91	1.50	19.71	88.45	1.96	15.45
2006	244.01	1.90	26.49	100.50	2.23	13.62
2007	348.53	2.71	42.83	128.22	2.85	27.58
2008	387.02	3.01	11.04	148.08	3.29	15.49
2009	431.84	3.36	11.58	167.20	3.71	12.91
2010	520.33	4.05	20.49	202.25	4.49	20.96
2011	649 00	5.06	24.73	228.57	5.07	13.01
2012	706.96	5.51	8.93	249.05	5.53	8.96
2013	809.96	6.31	14.56	293.00	6.51	17.65

## Data Availability

The data used to support the ﬁndings of this study are available from the corresponding author upon request.

## References

[1] Młodzianowski P., Młodzianowski D. (2018). Identification of areas of econophysical models application. *Economics*.

[2] Su B., Heshmati A. (2013). Analysis of the determinants of income and income gap between urban and rural China. *China Economic Policy Review*.

[3] Shen F., Liu W., Zhang S., Yang Y., Tao Shen H. (2015). Learning binary codes for maximum inner product search. *Proceedings of the IEEE International Conference on Computer Vision*.

[4] Shi L., Chuliang L. (2010). Re estimating the income gap between urban and rural households in China. *Procedia-Social and Behavioral Sciences*.

[5] Liu Y., Long C. (2021). Urban and rural income gap: does urban spatial form matter in China?. *Sage Open*.

[6] Li W., Wang X., Hilmola O. P. (2020). Does high-speed railway influence convergence of urban-rural income gap in China?. *Sustainability*.

[7] Li G. X. (2018). Convergence of income gap between new urbanization urban and rural residents—T-test method based on paired data. *Journal of Guizhou University of Finance and Economics*.

[8] Fong M. W. L. (2009). Digital divide between urban and rural regions in China. *The Electronic Journal on Information Systems in Developing Countries*.

[9] Beck T., Levine R. (2005). Legal institutions and financial development. *Handbook of New Institutional Economics*.

[10] Drèze J., Khera R. (2017). *Recent social security initiatives in India*.

[11] Zhang C., Zhu Y., Lu Z. (2015). Trade openness, financial openness, and financial development in China. *Journal of International Money and Finance*.

[12] Jalil A., Feridun M. (2011). The impact of growth, energy and financial development on the environment in China: a cointegration analysis. *Energy Economics*.

[13] Ji Q., Zhang D. (2019). How much does financial development contribute to renewable energy growth and upgrading of energy structure in China?. *Energy Policy*.

